# The interaction of 2D vortices in a developing pulsed plasma jet

**DOI:** 10.1038/s41598-025-31163-2

**Published:** 2025-12-07

**Authors:** Lei Dong, Kwing-So Choi, Yaxing Wang

**Affiliations:** 1 School of Mechatronics Engineering, Beijing Institute of Technology, Beijing, 100081 China; 2 Yangtze Delta Region Academy in Jiaxing, Beijing Institute of Technology, Jiaxing, 314019 China; 3 Faculty of Engineering, University of Nottingham, Nottingham, NG7 2RD UK

**Keywords:** Engineering, Physics

## Abstract

Two-dimensional vortex pairs were continuously generated by dielectric-barrier-discharge (DBD) pulsed plasma actuators, whose transformation to a free jet in the near field was investigated using a time-resolved particle image velocimetry (PIV) technique. At low modulation frequences, the plasma-induced vortex pairs developed into a free jet with little or no interactions. As the modulation frequency was increased, however, strong vortex interactions started taking place to initiate the leapfrogging, which continued every two plasma pulses. This helped sustain significant plasma-induced thrust further downstream. With a further increase in the modulation frequency, initial vortex pairs merged with a loss of jet thrust. In this study, these vortex interaction behaviours were carefully investigated using the wavelet spectral analysis as well as measured mean velocity and turbulent intensity profiles. Finally, the dynamics of leapfrogging vortex pairs and the mechanism of vortex merger initiation were investigated to depict the uniqueness in the 2D vortex interactions in a developing pulsed plasma jet.

## Introduction

 The dielectric barrier discharge (DBD) plasma actuators are a new type of flow control device without any moving parts, which have been used for many applications^1–5^ due to their unique characteristics. They have a very simple structure, consisting only of two electrodes separated by a dielectric sheet. When DBD plasma actuators are activated by high AC voltage, the air around the electrodes is ionized to form an ionic jet, which can transfer momentum to the surrounding air. The plasma discharge can be pulsed to form a series of pulsed jets, which can be tuned to the dominant frequency of fluid flow for control.

Jukes and Choi^6^ studied the effect of the pulsed plasma jet on the wake of a circular cylinder to suppress the vortex shedding. They showed a reduction in lift fluctuations of more than 70% with a drag reduction up to 32% when the plasma frequency was modulated to match that of vortex shedding. Here, narrowing of the wake was observed as the plasma promoted shear-layer roll-up. Greenblatt et al.^7^ carried out flow separation control of a flat-plate aerofoil using a pulsed plasma actuator, showing that the modulation frequency provided a lift enhancement. Here, two recirculation areas were formed over the suction surface due to plasma control, contributing to the increase in the lift force. Sato et al.^8^ investigated the influence of the modulation frequency of pulsed plasma actuators on the separation control of a NACA 0015 aerofoil using large-eddy simulation (LES). Lift and drag coefficients were significantly improved by plasma control when the modulation frequency was tuned to the separating shear-layer instability frequency, generating a short laminar separation bubble. The pulsed plasma actuator was also applied close to the flap shoulder of the NASA Energy Efficient Transport high-lift aerofoil model to mitigate the trailing-edge separation^9^. It was pointed out that the pulsed plasma should be actuated at the natural frequency of the trailing-edge flow field to modify the flow separation effectively.

In our recent flow control study of a small-aspect ratio wing using pulsed plasma actuators^10^, intriguing behaviours of two-dimensional (2D) vortex pairs were observed during the early stage of development, including the leapfrogging and vortex merger at certain plasma forcing conditions. Here, the leapfrogging was first studied by Helmholtz^11^, where two co-axial, co-rotating vortex rings periodically pass through each other. The behaviour of the leapfrogging was further examined by Mariani and Kontis^12^, demonstrating that the Reynolds number based on the orifice diameter and the vortex convection velocity were the dominant factors in the number of leapfrogs produced. Satti and Peng^13^ pointed out that the short interval between two vortex pairs prevented the formation of the leapfrogging due to the strong vorticity shedding from the trailing vortex pair. There were also experimental studied on the leapfrogging vortices involving starting jets^22,23^. A numerical study of leapfrogging carried out by Chen et al.^14^ confirmed the importance of the Reynolds number, the vortex core size and the vortices’ separation length. The dynamics and acoustics of two identical pairs of counter-rotating vortices were investigated using the compressible Navier-Stokes equations by Eldredge^15^, who showed that the pair of leapfrogging vortices eventually coalesced into a single counter-rotating pair of elliptical vortices after leapfrogging several times. The vortex merger typically involves an initial diffusive stage with limited interaction between vortices, followed by a convective stage characterised by significant deformation. This is subsequently followed by a secondary diffusive stage, where the induced velocity is too weak to further reduce the separation distance, and ultimately by a merged diffusive stage. Early theoretical studies of vortex merger^16,17^ identified a critical core-to-separation ratio above which two co-rotating vortices rapidly deform and merge into a single structure. This process was later refined by Meunier et al.^18^, who proposed a more robust merging criterion based on the second moment of vorticity. Experimental studies by Griffiths and Hopfinger^19^ and Meunier and Leweke^20^ confirmed similar critical values across various vorticity distributions.

To the best of the authors’ knowledge, this is the first report on the leapfrogging behaviour of vortex pairs generated by DBD plasma actuators. We are not aware of any studies on the leapfrogging of vortices in synthetic jets or pulsed jets. The main objective of this work is to elucidate the mechanisms where the modulation frequency of pulse plasma actuators affects the early interaction behaviour of vortex pairs in developing plasma jets. In this paper, we first present detailed observations made by time-resolved particle image velocimetry (PIV) on the initiation of the leapfrogging and merging process of vortex pairs generated by pulsed plasma actuators under different modulation frequencies. We then examine the interaction behaviour of vortex pairs against the plasma thrust distribution and the wavelet spectra. Development of mean velocity, turbulent intensity and the Reynolds stress is also investigated against the vortex movement. Finally, the dynamics of leapfrogging vortex pairs and the initiation of vortex merger are discussed, leading a map to classify different vortex interactions in terms of the circulation and the Reynolds number of the vortex pairs.

### Experimental set-up

**Fig. 1:**
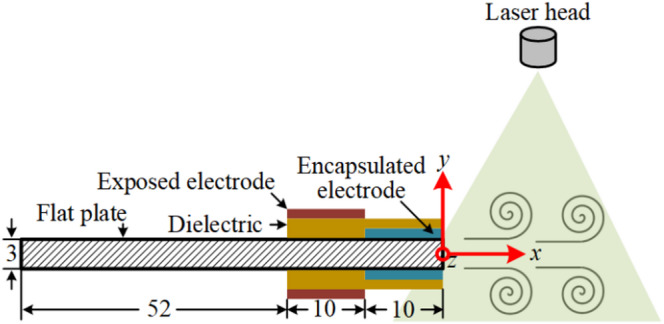
Sketch of the pulsed plasma actuators (not to scale) with a laser light sheet of the PIV system. All dimensions are in millimetres.

**Fig. 2:**
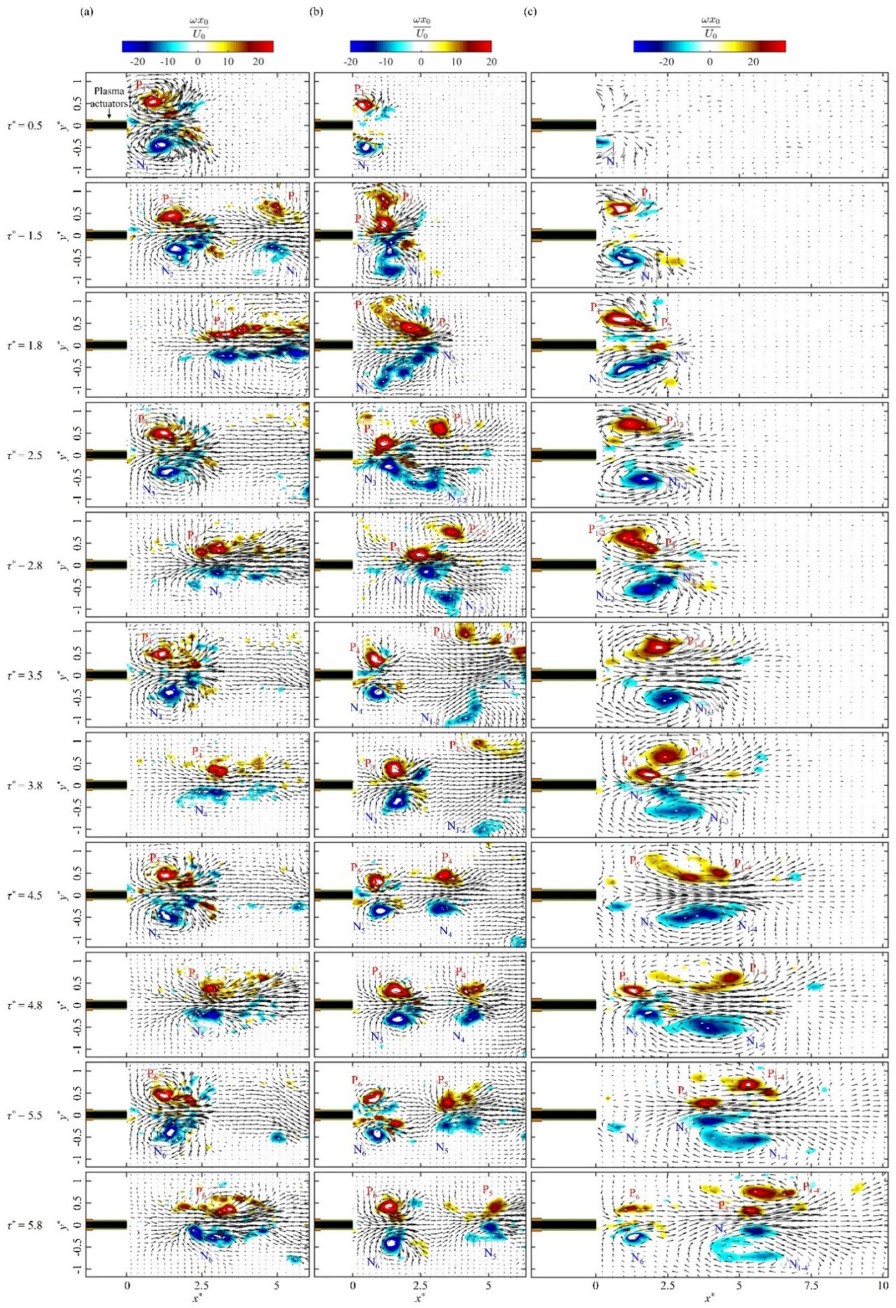
Instantaneous vorticity contours of initial vortex pairs with the velocity vectors after the activation of the pulsed plasma actuators: (**a**) case K, (**b**) case L and (**c**) case M.

The experiments were conducted at the University of Nottingham in a transparent, air-tight box of 0.6 × 0.3 × 0.3 m^3^, where the DBD pulsed plasma actuators were placed in the centre. This is a similar experimental setup with the same dimension employed by Whalley and Choi^27^, who investigated the behaviour of starting vortex from the plasma actuator in quiescent air. Here, the distance from the plasma actuator to the box wall is 5.2 times the vortex development region in streamwise direction (see Fig. 2), and 3.4 times in spanwise direction. Therefore, the box is large enough to allow the development of vortices without interference with the wall. Two-dimensional (2D) vortices were generated by the pulsed plasma actuators attached on both sides of a 3 mm-thick flat plate made of an aluminium composite panel comprising of a polyethylene core sandwiched by 0.3 mm-thick aluminium layers. This plate was supported by a 0.15 m-long strut. These plasma actuators consisted of exposed and encapsulated electrodes made of 0.05-mm thick copper tape with a dielectric sheet made of two layers of 0.15-mm thick Cirlex sheets. The *x*-coordinate is taken in the plasma-induced flow direction within the central plane of the plasma actuators, corresponding to the velocity component *U*. Likewise, the *y*-coordinate is taken normal to the plasma actuator with the velocity component *V* (see Fig. 1). The pulsed plasma actuators were excited in a sinusoidal waveform by a Minipuls 6 AC power supply whose peak-to-peak voltage amplitude *E*_*pp*_ was up to 60 kV with an operating frequency range from 5 to 20 kHz. In the present work, the excitation voltage was set between 14 kV and 18 kV at a fixed frequency of 7 kHz. The plasma actuators were pulse modulated by a square-wave signal of between 8 Hz and 160 Hz at a 50% duty cycle. This waveform was generated by the Field-Programmable Gate Array (FPGA) on a National Instruments (NI) CompactRIO controller. The excitation power supply, waveform generator and PIV system were synchronized by a timing box with a time delay of less than 5 ns.

Measurements of the velocity field induced by the pulsed plasma actuators were made using a time-resolved PIV technique. The test box was pre-seeded for each test with Di-Ethyl-Hexyl-Sebacat (DEHS) particles approximately 1 μm in diameter via a nozzle. We waited at least 10 min to make sure that the flow inside the box was in a quiescent condition before measurements. The test area was illuminated using a Litron LDY 302-PIV Nd: YLF dual-cavity laser with 15 mJ per pulse (see Fig. 1), whose laser sheet thickness was about 0.5 mm. Here, the time delay between two laser pulses was set to 150 µs. The image pairs were captured by a Phantom CMOS high-speed camera equipped with a 110 mm Canon lens with a resolution of 1280 × 800 at a sampling rate of 960 Hz for 4 s (0.5 s for plasma-off period, followed by 3 s of plasma-on and 0.5 s of resting periods). The PIV data was processed by Dantec DynamicStudio 2015a software using an adaptive PIV interrogation method, which automatically adjusted the size and shape of the interrogation area based on the local particle densities. The minimum and maximum interrogation areas were 8 × 8 and 32 × 32 pixels, respectively, with 50% overlap, generating about 16,000 velocity vectors in each frame. Data processing included the outlier detection analysis to replace spurious vectors with the median value of 3 × 3 neighbourhood vectors. The primary source of the PIV error was the uncertainty in the particle shifting distance (about 0.15 pixels) and dual-laser interval time (negligible in the present study), resulting in a total error of PIV measurements less than 5%. A more detailed uncertainty analysis of this PIV system can be found in Dong et al.^24^. Based on the measured velocity fields, the vortex identification was made using the *λ*_2_-criterion^25^, where the vortex core region was determined by applying the threshold value of *λ*_2_ = −10,000. The location of the minimum *λ*_2_ value within this region was then identified as the vortex centroid. A similar methodology was previously used by Dong et al.^10,24^ to identify the vortex centroid of wing-tip vortices.

## Results and discussion

### Initial development of vortex pairs

After a preliminary test of plasma activation parameters, three cases K, L and M with different vortical interactions are identified for further investigation. This was carried out no fewer than three times in each case, all of which produced consistent results as summarised in Table 1. Here, the peak-to-peak excitation voltage and modulation frequency of the pulsed plasma actuators are given by *E*_*pp*_ and *f*_*m*_, respectively, while its momentum coefficient is calculated as *C*_*µ*_ = *F*/(*ρU*_0_^2^*y*_0_) when the starting vortex pair initiated by the first plasma pulse reaches its maximum circulation *Γ*_0_ with the spanwise spacing *y*_0_ between the pair at the streamwise distance *x*_0_ in time *t*_0_, where *ρ* is the air density and *U*_0_ is the convection velocity of the starting vortex pair. *F* is the plasma thrust per unit depth^6^ given by $\:F=\int\:\rho\:{U}^{2}\text{d}y$, which is computed by integrating the *U*-component velocity over y^*^ = −1 and 1, where y^*^ is the non-dimensional coordinate defined by y^*^ = *y*/*y*_0_. Similarly, x^*^ is given by x^*^ = *x*/*x*_0_.

**Table 1:** Plasma excitation conditions.

Cases	E_pp_ (kV)	f_m_ (Hz)	U_0_ (m/s)	x_0_ (mm)	y_0_ (mm)	F (*N*/m)	C_µ_
case K	18	20	0.39	8.9	20.5	0.093	24.83
case L	18	40	0.42	8.5	19.1	0.057	14.09
case M	18	80	0.20	5.3	17.2	0.031	37.52

The detailed evolution of initial vortex pairs is presented in Fig. 2, depicting their non-dimensional vorticity *ωx*_0_*/U*_0_ and the velocity field with velocity vectors. Here, non-dimensional time is defined by τ^*^ = *t*⋅*f*_*m*_. P/N in Fig. 2 indicates the vortex pair with positive/negative vorticity, while the vortices resulting from the merger of the m-th to n-th vortex pairs are denoted as P_m−n_ and N_m−n_, respectively. The first counter-rotating vortex pair for case K is observed at τ^*^ = 0.5 after the activation of the pulsed plasma actuators. As the vortex pair moves downstream, the circulation of vortices increases, thereby increasing the induced velocity. This strains the vortices to break up into a chain of vortices, see Fig. 2a at τ^*^ = 1.8, 2.8, 3.8, 4.8 and 5.8. For case L, the first vortex pair P_1_/N_1_ can only manage to move for a short distance (x^*^ < 1) in an initially quiescent flow. This strongly affects the movement of the following vortex pair P_2_/N_2_, allowing it to go through the first vortex pair P_1_/N_1_ as shown in Fig. 2b at τ^*^ = 1.5. After that, vortex pairs P_1_/N_1_ and P_2_/N_2_ start merging to form a single vortex pair P_1 − 2_ and N_1 − 2_ in Fig. 2b at τ^*^ = 2.5. A similar vortex interaction is observed with the subsequent plasma pulses, where the third vortex pair P_3_/N_3_ leapfrogs P_1 − 2_/N_1 − 2_. Thereafter, we observe an increase in the distance between the vortex pairs at τ^*^ = 3.5, allowing an interaction-free development of vortex pairs P_4_/N_4_ and P_5_/N_5_ without leapfrogging. Another type of vortex interaction is a vortex merger (case M), where the trailing vortex pair catches up with the leading pair and merges with it, see Fig. 2c at τ^*^ = 1.8, 2.8 and 4.5, which is similar in behaviour to the co-rotating vortex merger^28,29^. As a result, the first four vortex pairs merge together to form strong vortex pairs P_1 − 2_/N_1 − 2_, P_1 − 3_/N_1 − 3_ and P_1 − 4_/N_1 − 4_. The only leapfrogging event for case M is observed when the fifth vortex pair P_5_/N_5_ leapfrogs P_1 − 4_/N_1 − 4_ at x^*^ = 6 and τ^*^ = 5.8. These results suggest that the leapfrogging can be induced by plasma actuators at a low momentum coefficient, as shown in Table 1.

**Fig. 3:**
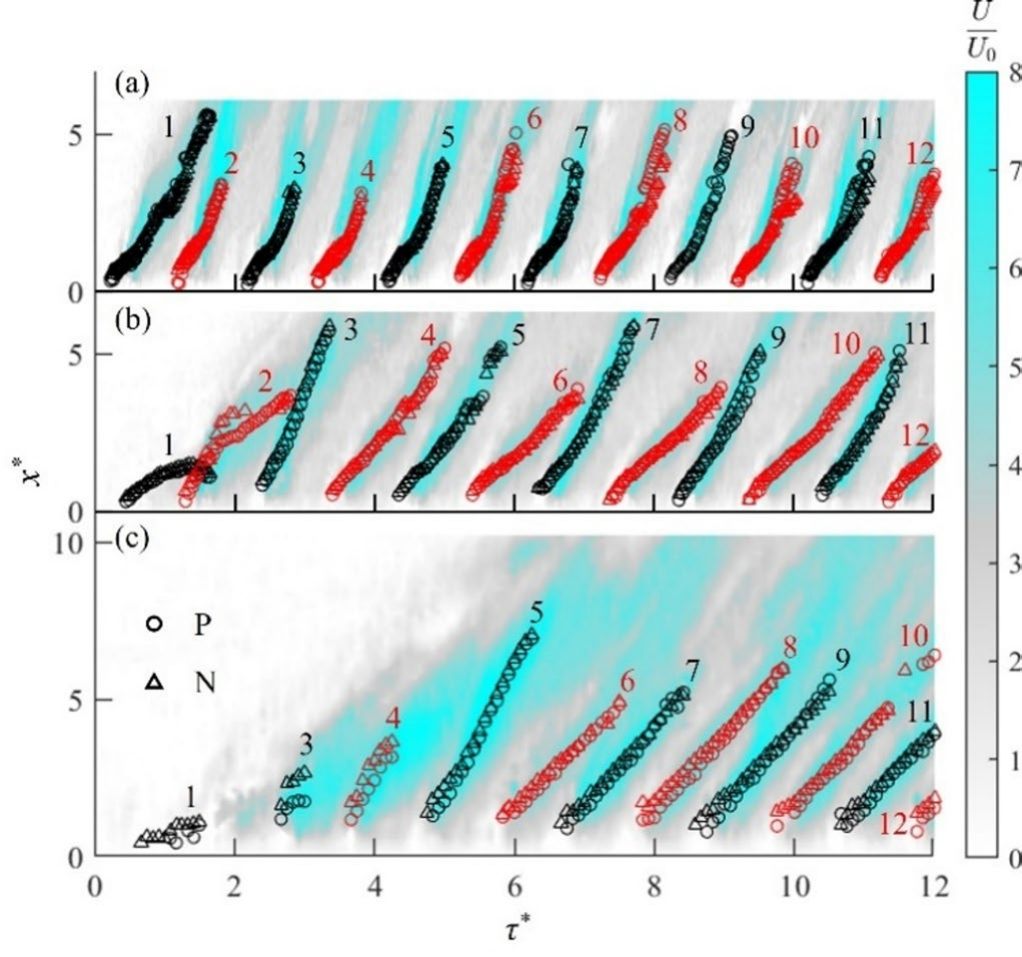
Movement of initial vortex pairs as a function of time after the plasma activation at τ^*^ = 0, superimposed the *U*-component velocity on the centreline: (**a**) case K, (**b**) case L and (**c**) case M. The vortices with positive vorticity are indicated by the circles, while the vortices with negative vorticity are denoted by the triangles.

To further investigate the interactions of vortex pairs generated by the pulsed plasma actuators with different modulation frequencies, the streamwise locus x^*^ of initial 12 vortex pairs is presented as a function of τ^*^ in Fig. 3 for cases K, L and M, superimposed by the non-dimensional velocity *U/U*_*0*_ (shown in colour) along the centreline (y^*^ = 0). Figure 3 shows that each vortex pair moves along the region of maximum *U*-component velocity. It also shows that the vortices undergo a development stage for about 100 ms, lasting for two plasma pulses (τ^*^ = 2) for case K, 4 pulses (τ^*^ = 4) for case L, and 8 pulses (τ^*^ = 8) for case M. This development stage is followed by a periodic stage, during which the repeatable movement of vortices is observed.

At a low plasma modulation frequency of *f*_*m*_ = 20 Hz for case K, the first pair of vortices travels far downstream (x^*^ > 3.5). Then, the second pair is produced, resulting in a weak interaction between the two vortex pairs. Therefore, the vortex pairs for case K initiate a periodic stage as soon as they are generated as shown in Fig. 3a. As *f*_*m*_ is doubled to 40 Hz for case L, the first vortex pair P_1_/N_1_ moves only for a short distance in quiescent air before it is caught up by the second vortex pair P_2_/N_2_. The second vortex pair then passes through the first vortex pair at x^*^ = 1.5 to make a leapfrogging. The backward movement of the first vortex pair in Fig. 3b is a result of this leapfrogging. A similar behaviour is observed for the third vortex pair P_3_/N_3_, which leapfrogs the merged vortex pairs of P_1_/N_1_ and P_2_/N_2_ at x^*^ = 3.5. As the third vortex pair P_3_/N_3_ moves away from the actuators (x^*^ > 5), the subsequent vortex pairs P_4_/N_4_ and P_5_/N_5_ move downstream without interaction. Then, the following two vortex pairs P_6_/N_6_ and P_7_/N_7_ form leapfrogging, see Fig. 3b, which continues between the subsequent pairs P_8_/N_8_ and P_9_/N_9_, etc., indicating that they evolve into the periodic stage of continuous leapfrogging. With a further increase in the plasma modulation frequency to *f*_*m*_ = 80 Hz for case M, quite a different vortex interaction takes place. Here, the vortex merger occurs between the vortex pairs from the start of the plasma actuation, which is the reason why the second pair is not visible in Fig. 3c. The vortex merger continues until the fifth vortex pair before a leapfrogging of P_5_/N_5_ through the merged vortex pairs. The loci of subsequent vortex pairs are parallel to each other, see Fig. 3c, indicating that case M quickly reaches the periodic stage.

**Fig. 4:**
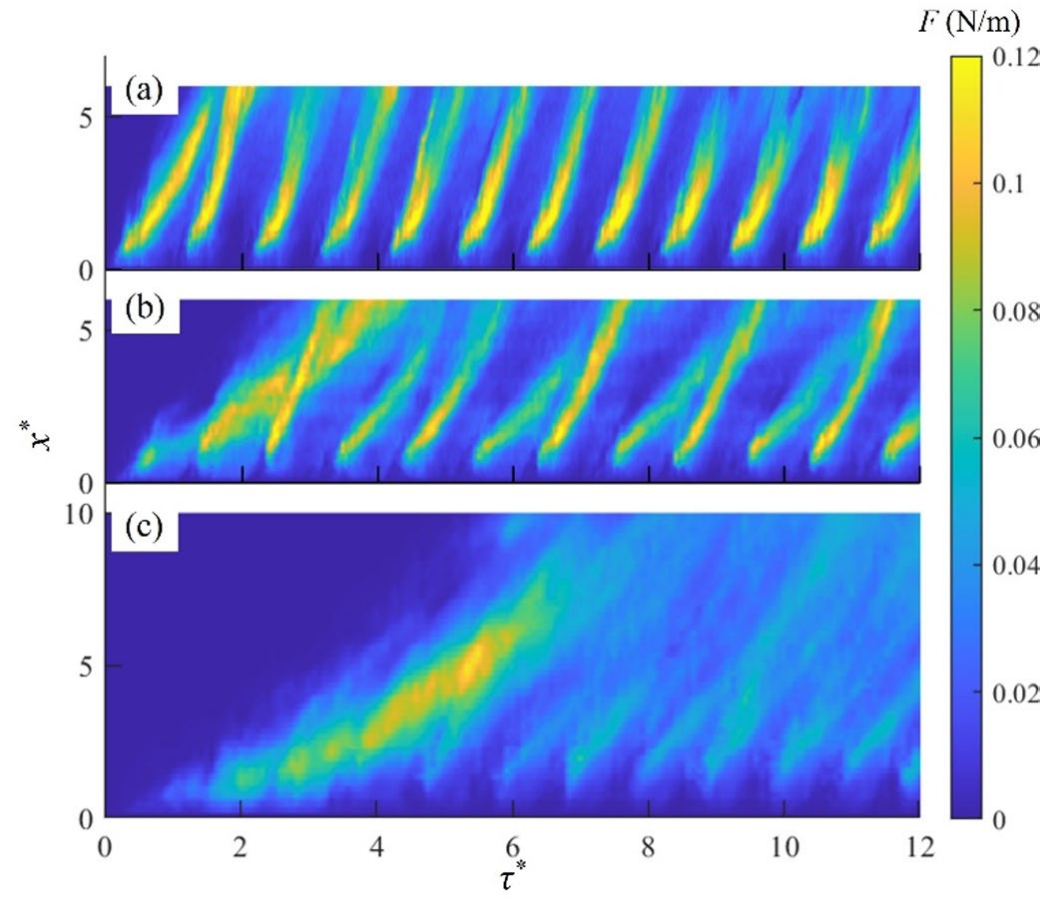
Plasma thrust *F* distributions of initial 12 pulsed jets: (**a**) case K, (**b**) case L and (**c**) case M.

The thrust force *F* of the plasma jet in the initial 12 pulses is shown in Fig. 4 as a function of τ^*^ and x^*^ for three cases. For case K, a significant plasma thrust is observed at the plasma initiation (τ^*^ < 2) and near the plasma actuators (x^*^ < 3.5). However, it decreases rapidly downstream due to a reduction in convection velocity of the vortex pair, as shown in Fig. 3a. High thrust force is also observed for case L at early times (τ^*^ < 4), which is driven by the interaction of the initial three vortex pairs. Subsequently, the high thrust force appears in every two pulses downstream (x^*^ > 4). This is due to the leapfrogging, enabling the trailing vortex pairs to gain the momentum from the leading vortex pairs, see Fig. 2b. In contrast, a significant thrust is generated only once for case M. This is due to the merger of early vortex pairs, which is leapfrogged by the fifth vortex pair. However, this thrust is reduced quickly as the plasma pulsed jet reaches the periodic stage. It should be noted that the pulse duration is reduced with an increase in the modulation frequency, therefore the thrust per pulse of cases L and M is half and a quarter of that of case K, respectively. Figure 4 also indicates that the plasma jet forcing can be sustained far downstream when vortex pair interactions similar to case L occur, suggesting that high flow control efficiency may be achieved by tuning the modulation frequency of the plasma actuators to promote the leapfrogging.

**Fig. 5:**
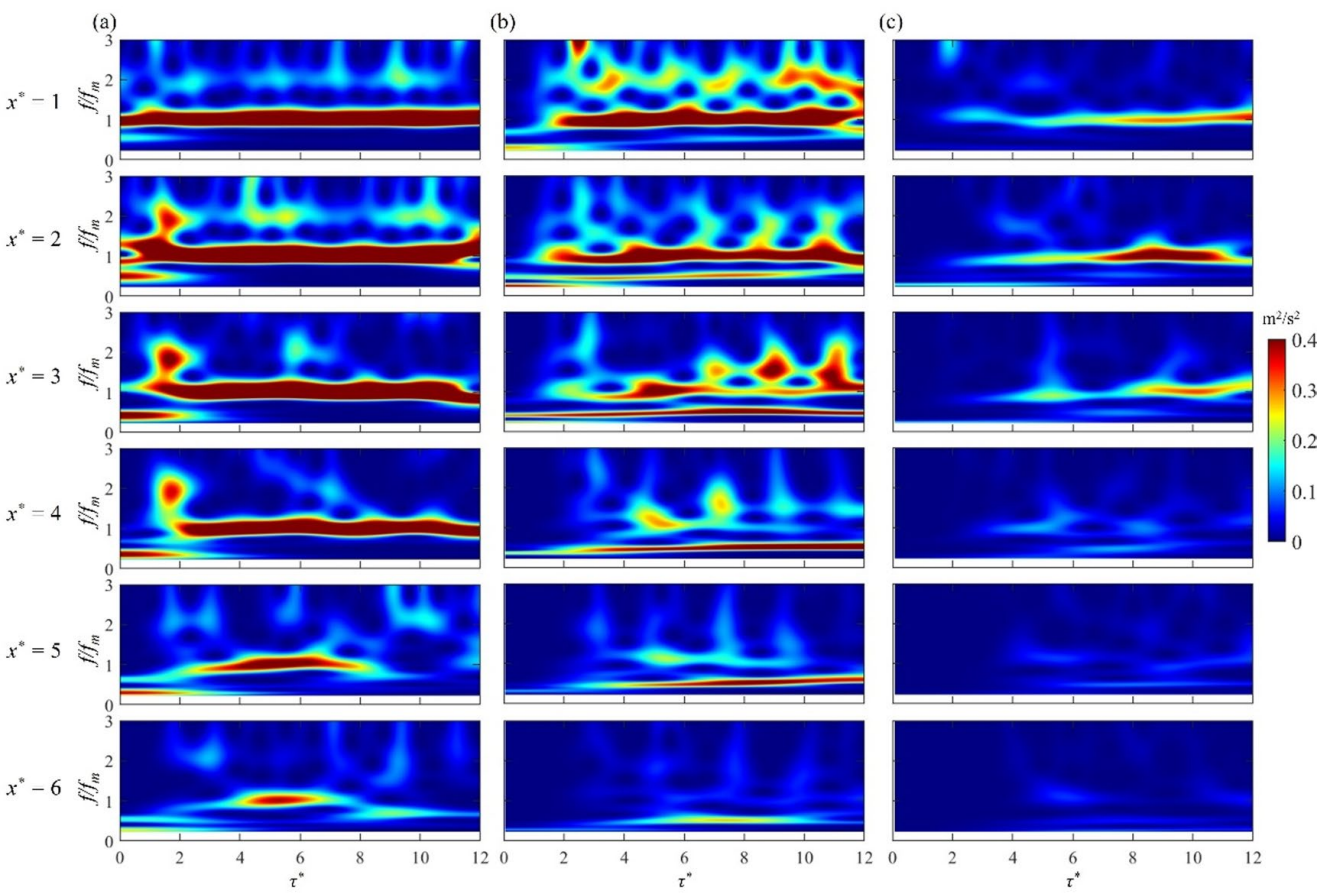
Wavelet spectra of fluctuation velocity *u* for the initial 12 pulses at various streamwise positions at y^*^ = 0: (**a**) case K, (**b**) case L and (**c**) case M.

Figure 5 presents the wavelet spectra of the streamwise fluctuating velocity *u* along the centreline (y^*^ = 0) at various streamwise positions from x^*^ = 1 to 6 for cases K, L and M, where the frequency *f* is normalized by the modulation frequency *f*_*m*_ of the plasma actuators. All cases are shown to exhibit dominant spectral energy at the primary frequency at *f*/*f*_*m*_ = 1, indicating that the induced vortex evolution remains phase-locked with the plasma pulse. The modulation-frequency-dominated wavelet energy gradually decays as a result of progressive transfer of energy from the organized vortex structures to the mean flow in a similar way as the development of synthetic jets^26^. For case K, the emergence of high-frequency energy between x^*^ = 2 and 4 at the start of plasma jet development (τ^*^ < 2) is due to small broken-up vortices during the initial vortical interactions. These vortices are then clustered in a long chain, see Fig. 2a, giving rise to lower-frequency energy. For case L, subharmonic frequencies begin to emerge at x^*^ = 1, signifying the onset of leapfrogging as observed in Fig. 2b. With a development of the leapfrogging, there is a transfer of spectral energy from the fundamental frequency to subharmonics. Due to the merger of multiple vortex pairs for case M, a significant spectral energy is only observed between x^*^ = 1 and 3.

**Fig. 6:**
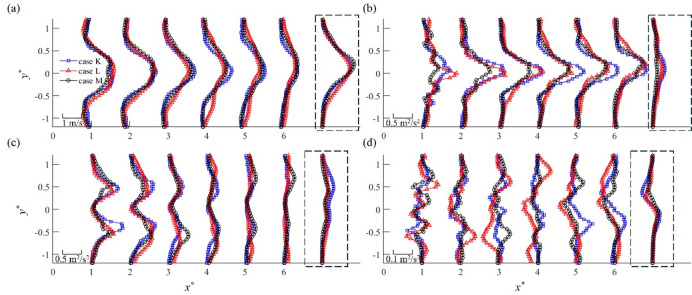
Initial development of the pulsed plasma jet: (**a**) mean velocity profiles, (**b**) turbulent intensity profiles of fluctuation velocity *u*, (**c**) turbulent intensity profiles of fluctuation velocity *v*, and (**d**) the Reynolds stress profiles. Corresponding profiles from the periodic stage of the jet at x^*^ = 6 are shown on the right side of each subfigure (encircled by dash lines) for comparison.

The jet flow development from the pulsed plasma actuators is detailed in Fig. 6, where the mean velocity, streamwise and spanwise turbulent intensities and the Reynolds stress profiles are presented for three cases. These results are obtained using the first two plasma pulses (τ^*^ = 2) for case K, 4 pulses (τ^*^ = 4) for case L, and 8 pulses (τ^*^ = 8) for case M, corresponding to the development stage of the pulsed plasma jet as shown in Fig. 2. For comparison, the profiles in the periodic stage at x^*^ = 6 are included on the right side of each subfigure. Figure 6a shows a similar development of mean velocity profiles in each case, where the peak velocity gradually decreases downstream. Compared to the periodic-stage profiles, the plasma jets in the development stage exhibit much lower mean velocities as the jets travel through quiescent air. Notably, case L exhibits higher mean velocity at the outer edges of the jet (y^*^ > ±0.5) between x^*^ = 3 and 6 as compared to those for cases K and M. A similar increase in the *u-*component turbulent intensity is observed for case L at these locations. These are attributed to the leapfrogging behaviour of vortex pairs for case L, where the outward motion of the leading vortex pairs enhances the momentum transport across the jet. Figure 6b shows that the streamwise turbulence intensity $\:\stackrel{-}{{u}^{2}}$ in the development stage is considerably higher than that in the periodic stage. Again, this is due to quiescent flow at the start of plasma activation, making the plasma jet vortex-dominant with lower convection velocity. In contrast, the spanwise turbulence intensity $\:\stackrel{-}{{v}^{2}}$ decreases downstream, as shown in Fig. 6c, reaching to the profile similar to that of the period stage. As expected, the twin peaks of $\:\stackrel{-}{{v}^{2}}$ are observed at each centre of the pair of vortices (y^*^ = ±0.5), where the maxima and minima of the Reynolds stress are obtained, see Fig. 6d. A greater Reynolds stress $\:-\stackrel{-}{uv}$ distribution for case L between x^*^ = 3 and 6 is a result of strong vortex-vortex interactions during the leapfrogging process, suggesting a potential enhancement of flow control effectiveness by the leapfrogging at an early stage of the pulsed plasma jet actuation.

### Dynamics of leapfrogging

**Fig. 7:**
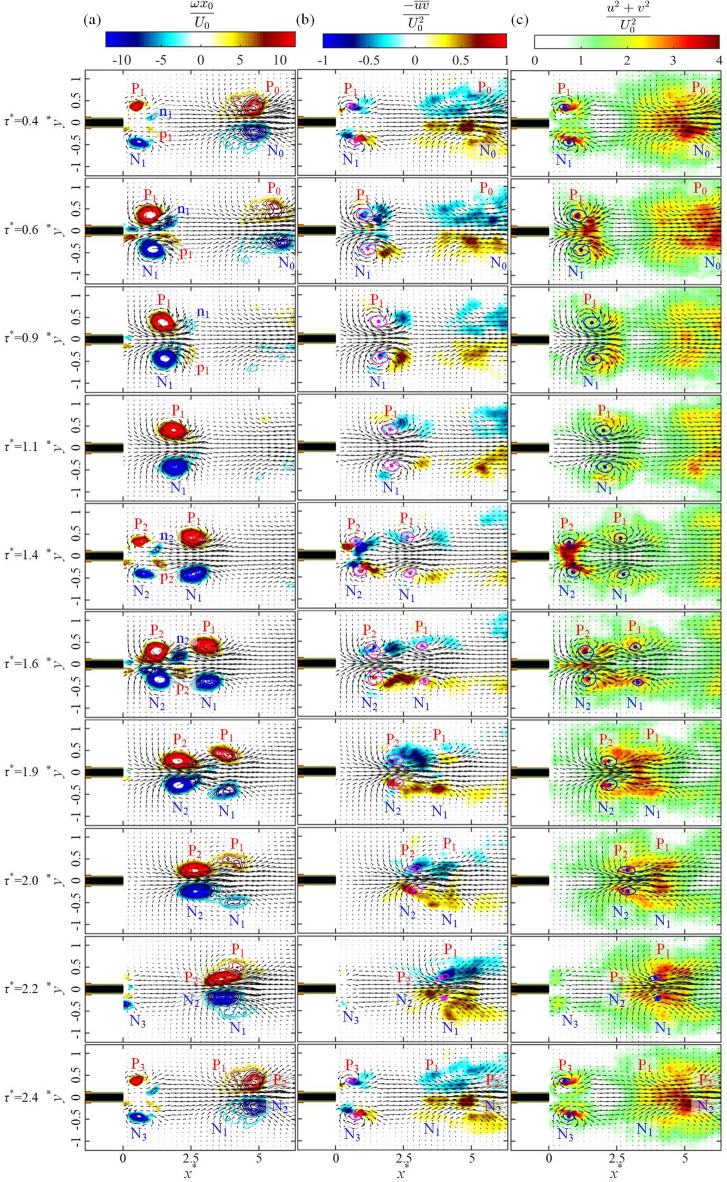
The distribution of (**a**) the phase-averaged vorticity, (**b**) the Reynolds stress, and (**c**) the TKE during the periodic leapfrogging stage for case L. The cores and centroids of the vortices in the Reynolds stress and turbulent intensity plots are shown in magenta and blue circles and dots, respectively.

As shown in Figs. 2 and 3, plasma-induced vortex pairs for case L are seen to interact with each other every two pulses of plasma actuation, where the trailing vortex pair can overtake and leapfrog the leading pair. Here we investigate the dynamic behaviour of this leapfrogging process for one cycle between τ^*^ = 0.4 and 2.4. Figure 7 shows the phase-averaged flow quantities superimposed by the velocity vectors, including (a) the vorticity ω*x*_0_/*U*_0_, (b) the Reynolds stress $\:-\stackrel{-}{uv}/{U}_{0}^{2}$ and (c) the turbulent kinetic energy (TKE) $\:{(u}^{2}+{v}^{2})/{U}_{0}^{2}$, which are all nondimensionalised by the convection velocity *U*_0_.

The first frame (τ^*^ = 0.4) of the figure coincides with the emergence of the first primary vortex pair P_1_ and N_1_ and the associated secondary vortices p_1_ and n_1_, as the previous pair of vortices P_0_ and N_0_ moves away from the field of view. It should be noted that these secondary vortices are generated to preserve the no-slip boundary condition during the plasma activation. The first primary vortex pair P_1_ and N_1_ is seen to grow in Fig. 7a, increasing its vorticity further at τ^*^ = 0.6 when it is wrapped around by the secondary vortices n_1_ and p_1_. It moves slowly, arriving at x^*^ = 2.5 at τ^*^ = 1.4, when the second vortex pair P_2_ and N_2_ emerges from the plasma actuators at x^*^ = 0.7, to be wrapped around by the secondary vortices n_2_ and p_2_. The induced velocity of the first vortex pair P_1_ and N_1_ helps the second vortex pair P_2_ and N_2_ accelerate its movement (τ^*^ = 1.4 to 1.9), catching up and leapfrogging P_1_ and N_1_ (τ^*^ = 2.2 to 2.4). As the vortex pair P_2_ and N_2_ passes through P_1_ and N_1_, the vortex pairs P_2_/N_2_ and P_1_/N_1_ merge and move away (x^*^ = 5.5 at τ^*^ = 2.6). This completes one cycle of the leapfrogging process in the periodic stage, which will repeat for the following vortex pairs.

The corresponding Reynolds stress distribution is presented in Fig. 7b, where the vortex cores and their centroids, which are identified by the minimum value of *λ*_2_, are indicated by magenta circles and dots, respectively. Here, some of weakened vortex cores (e.g., P_1_/N_1_ from τ^*^ = 1.9 to 2.4 and P_2_/N_2_ from τ^*^ = 2.2 to 2.4) are absent due to the difficulty in identification. The negative Reynolds stress is observed downstream of the positive vortex P_1_ at τ^*^ = 0.6 and P_2_ at τ^*^ = 1.4 as a result of the positive *u* and *v* velocities. Likewise, the positive Reynolds stress region is seen downstream of the negative vortices N_1_ and N_2_ due to the positive *u* and negative *v* velocities. There is a further increase in the Reynolds stress at the start of the leapfrogging process (τ^*^ = 1.9) when the second vortex pair P_2_/N_2_ comes closer to the first vortex pair P_1_/N_1_.

Figure 7c presents the distribution of TKE during the leapfrogging process, where the *λ*_2_-identified vortex cores are shown by blue circles for clarity. The evolution of TKE closely mirrors that of the Reynolds stress as shown in Fig. 7b since TKE is mainly produced by the Reynolds stress. In other words, the high TKE is observed at the location where the Reynolds stress is high. Specifically, the high level of TKE is observed downstream of the initial pair of vortices P_1_/N_1_ at τ^*^ = 0.4, gradually intensifying until τ^*^ = 0.6. The substantial TKE is found with the formation of the second vortex pair P_2_/N_2_ at τ^*^ = 1.4, and later between the two vortex pairs at τ^*^ = 1.9.

### Initiation of vortex merger

**Fig. 8:**
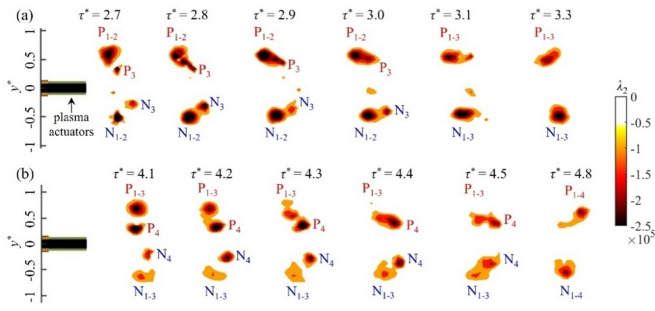
The merging process of successive vortex pairs for case M: (**a**) the first and second vortices merging with the third vortex pair and (**b**) the first to third vortices merging with the fourth vortex pair. The vortices are identified by isocontours of *λ*_2_.

The merger process of vortex pairs during the pulsed plasma actuation is studied in this section. Figure 8 shows a series of vortex interaction for case M, where the trailing vortex pairs P_3_/N_3_ and P_4_/N_4_ move downstream of the leading pairs P_1 − 2_/N_1 − 2_ (at τ^*^ = 2.7) and P_1 − 3_/N_1 − 3_ (at τ^*^ = 4.2), respectively, in their induced velocity field. With an increased modulation frequency, the time interval between successive vortex pairs is reduced, so the trailing vortex pairs are underdeveloped with weak circulation. Therefore, their evolution is strongly influenced by the leading vortex pairs, resulting in a merger of co-rotating vortex cores. Here, the leading and trailing vortices rotate in the same direction, causing an inward movement of the leading pair and an outward movement of the trailing pair in the spanwise direction. Meanwhile, the vortex cores deform from circular to elliptic shape due to mutual interaction. Eventually, a complete merger occurs with the formation of a single vortex^28^.

**Fig. 9:**
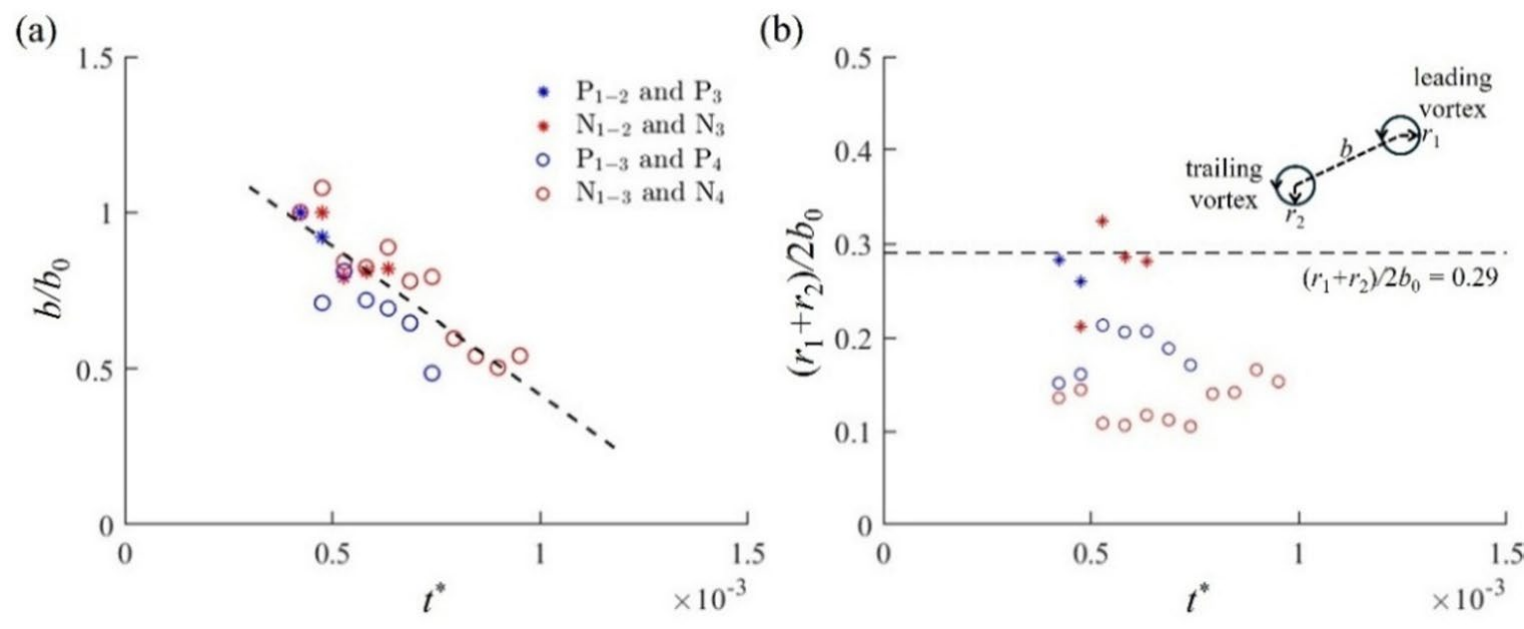
The evolution of (**a**) the separation distance and (**b**) the core radius of the merging vortex pairs for case M, where the vortex position is schematically illustrated.

To study the merging process of asymmetric vortex pairs as seen in Fig. 8, the evolution of the non-dimensional separation distance *b/b*_*0*_ and the average core radius of merging vortex pairs (*r*_1_ + *r*_2_)/2*b*_0_ for case M is plotted as a function of non-dimensional time t^*^ in Fig. 9. Here, the non-dimensional time is given by t^*^ *= tv/y*_0_^2^ as suggested by Carretela and Williamson^29^, where *t* is the dimensional time, *v* is the kinematic viscosity of air and *y*_0_ is the spanwise spacing of the vortex pair P_1_/N_1_. The initial separation distance between the two co-rotating vortices is *b*_0_. The linear behaviour of *b/b*_0_ with time t^*^ in Fig. 9a suggests that the asymmetric vortex pairs directly enter the convective merging stage without going through the initial diffusive stage which is typically observed for equal-strength vortices^29^. This is further demonstrated by the development of the average core radius (*r*_1_ + *r*_2_)/2*b*_0_ in Fig. 9b, where *r*_1_ and *r*_2_ denote the radii of the leading and trailing vortices, respectively. The average core radius (*r*_1_ + *r*_2_)/2*b*_0_ of merging vortex pairs for case M is much smaller than the value of 0.24 found by Meunier et al.^18^ or 0.29 by Cerretelli and Williamson^29^, suggesting that the merger of vortices with unequal strength in the present case takes place much quickly than those with equal strength. This is in agreement with Brandt and Nomura^30^, who concluded that the disparities in circulation and core size reduce the effective critical threshold for merger as the weaker vortex being more susceptible to rapid distortion and absorption by the stronger one.

### Effect of modulation frequency

**Fig. 10:**
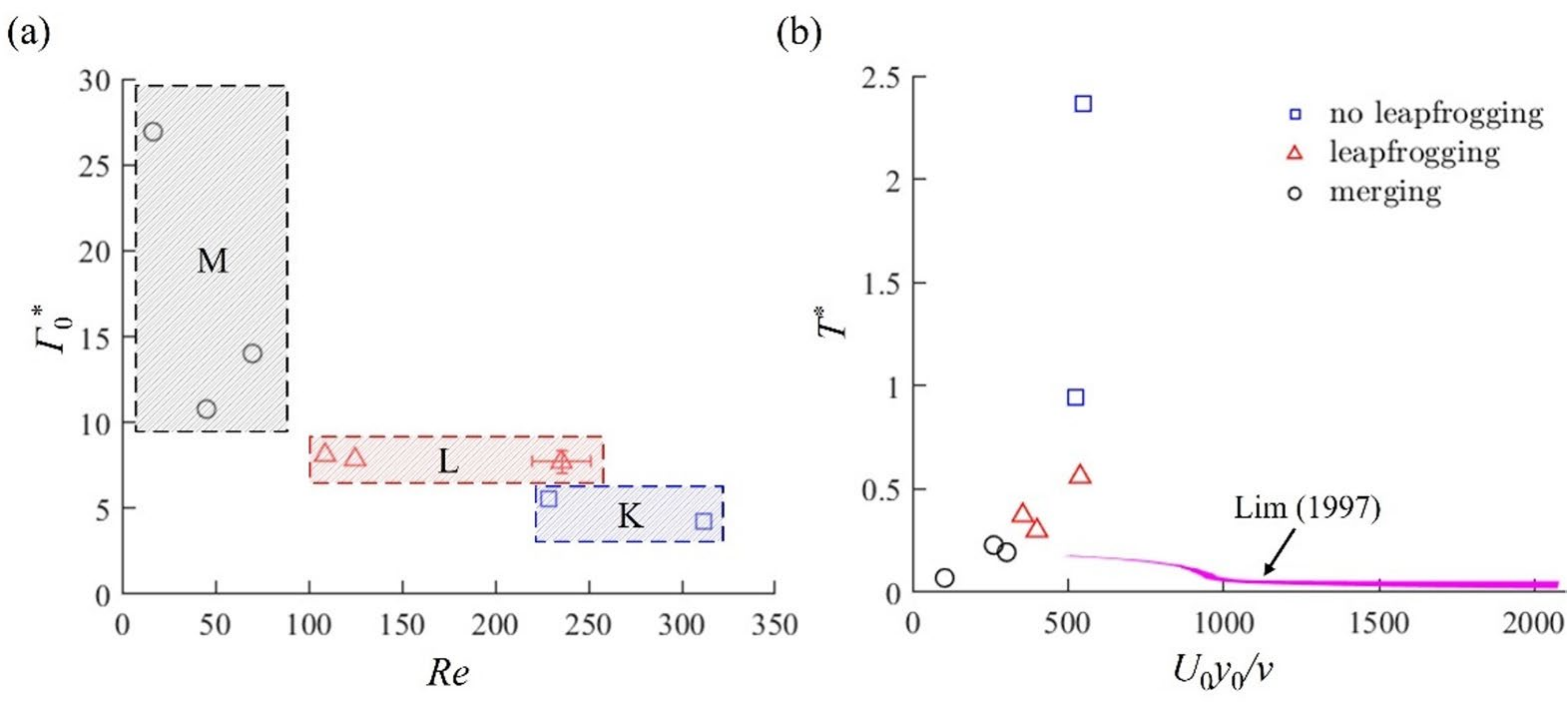
The vortex interaction map based on (**a**) the circulation *Γ*_0_^*^ and the Reynolds number *Re* of the initial vortex pair, and (**b**) the Reynolds number *U*_0_*y*_0_/*ν* and the nondimensional time interval of the vortices T^*^ proposed by Lim^21^, where the purple region denotes the conditions successfully forming the leapfrogging between two vortex rings.

Considering the strong influence of the vortex strength and the Reynolds number on the behaviour of vortex interaction, we can map the occurrence of cases K, L and M during the pulsed plasma actuation based on the non-dimensional circulation of the vortex pair *Γ*_0_^*^ = *Γ*_0_/(*U*_0_* × *_0_) and the Reynolds number *Re* = *x*_0_*U*_0_/*ν* as shown in Fig. 10a. When the circulation of the initial vortex pair *Γ*_0_^*^ is large (*Γ*_0_^*^ > 6), a strong interaction between the vortex pairs is expected. Then, if the Reynolds number is small (*Re* < 100) at the same time, this results in a merger of vortex pairs (case M). If the Reynolds number is large (*Re* > 100), on the other hand, the leapfrogging can take place (case L). When the circulation of the initial vortex pair *Γ*_0_^*^ is small (*Γ*_0_^*^ < 6), the vortex interaction is not strong enough to cause merger or leapfrogging (case K), which is consistent with the numerical simulation results by Chen et al.^14^ who showed that no leapfrogging of vortices could take place without sufficient circulation.

We can compare the behaviour of two-dimensional vortices generated by pulsed plasma actuators with that of piston-generated vortices. Lim^21^ suggested that the condition for leapfrogging is determined by the time interval between successive vortex rings and their Reynolds number based on the piston diameter. Figure 10b compares the present results with those reported by Lim^21^ for the piston-generated vortex rings. In our study, the spanwise distance *y*_0_ of the initial vortex pair can be considered as the effective diameter of the piston, while 1/*f*_*m*_ represents the time interval between adjacent vortex pairs. Therefore, the non-dimensional time interval between successive vortex pairs can be given by T^*^ = *U*_0_/(*f*_*m*_*y*_0_), while the Reynolds number is given by *Re* = *U*_0_*y*_0_/*ν*. Here, Lim^21^ observed the leapfrogging of vortex rings at the non-dimensional time interval of T* = 0.02 ~ 0.06 for the Reynolds number of *Re* = 1050 ~ 2080. For a reduced Reynolds number of *Re* = 480, the leapfrogging was only found at T* ~ 0.18. On the other hand, the present results indicate that the leapfrogging takes place at T* = 0.3 ~ 0.6 for *Re* = 300 ~ 550. Therefore, our value of T* is much greater than that of Lim^21^ but is in good agreement with Satti and Peng^13^, who obtained T* ~ 0.55 for *Re* = 1550. As the non-dimensional time interval is reduced, we found that the distance between the vortex pairs is reduced, and the vortices start merging rather than leapfrogging. Satti and Peng^13^ also observed a similar vortex interaction behaviour at small values of T*, where the strength of the second vortex is reduced through vorticity shedding due to an increased interaction with the first vortex. Consistent with Lim’s condition for leapfrogging (shown in purple colour), our results demonstrate that the time interval T^*^ must be large enough for the leapfrogging to take place if the Reynolds number *U*_0_*y*_0_/*ν* of the vortex pairs is small. We should note, however, that there is a clear difference in the flow field where the vortices are generated between the present study and the previous studies^13,21^. In other words, the 2D vortices in the present study are generated by the wall jets developed over the DBD plasma actuators, while vortex rings are the result of roll-up of the shear layer created by the “slug flow” during the piston motion^13,21^. Therefore, the criteria for the leapfrogging are expected to be quite different between these studies despite of a qualitative agreement.

## Conclusions

When we activated DBD pulsed plasma actuators, 2D vortex pairs were continuously generated to go through different interaction behaviours depending on the plasma modulation frequency. Here we investigated the behaviour of these vortex pairs in the near field using a time-resolved PIV technique. At a low plasma modulation frequency of 20 Hz, no or very little interactions were observed between the pairs of vortices. It was found that the developing stage of plasma jets exhibited much lower mean velocities than the final stage of development. However, the streamwise turbulence intensity of the plasma jets was considerably higher than that in the periodic stage. This is because the plasma jets are more vortex-dominant at the start of plasma activation with a low convection velocity. In contrast, the spanwise turbulence intensity of the plasma jets reduced downstream quickly to reach the final-stage profile. As expected, the twin peaks the spanwise turbulent intensity were observed at each centre of the moving pair of vortices, where the maxima and minima of the Reynolds stress were shown.

As the plasma modulation frequency was doubled, strong vortex interactions started taking place to initiate the leapfrogging. Here, the first vortex pair from the plasma actuators moved only for a short distance in the quiescent air before it was caught up by the second vortex pair. A similar leapfrogging behaviour was observed for the third vortex pair, which leapfrogged the merged first and second vortex pairs. As these vortex pairs moved away from the actuators, the subsequent two vortex pairs moved downstream without interaction. Then, the next two vortex pairs (6th and 7th) formed the leapfrogging, which was followed by another leapfrogging between the 8th and 9th vortex pairs, etc. to evolve into the periodic stage of the continuous leapfrogging process for every two vortex pairs. Our results indicated that a high thrust force appeared in every two plasma pulses, which sustained downstream when leapfrogging of vortex pairs took place, suggesting that high flow control efficiency may be achieved by tuning the modulation frequency of the plasma actuators to promote the continuous leapfrogging.

With a further increase in the plasma modulation frequency, the vortex merger took place among the initial vortex pairs. This is due to the reduced time interval between successive vortex pairs, so the trailing vortex pairs were underdeveloped. Therefore, the evolution of vortex pairs was strongly influenced by the leading vortex pairs, resulting in a merger of co-rotating vortex cores. It was shown that the unequal vortex pairs from the plasma actuators merged more quickly than the co-rotating vortex pairs of equal strength. In this case, a significant thrust is generated only at the start of plasma initiation, which is reduced quickly towards the final periodic stage with no further interactions between the vortices.

Finally, we presented a map to show the occurrence of these vortex interactions based on the non-dimensional circulation of the vortex pair and the Reynolds number. When the circulation of the initial vortex pair is sufficiently large, a strong interaction between the vortex pairs is expected. Then, if the Reynolds number is small at the same time, this results in a merger of vortex pairs. If the Reynolds number is large, on the other hand, the leapfrogging can take place. When the circulation of the initial vortex pair is small, the vortex interaction is not strong enough to cause merger or leapfrogging.

## Data Availability

The datasets generated during and/or analysed during the current study are available from the corresponding author on reasonable request.
